# Investigation of Microcrack Propagation and Energy Evolution in Brittle Rocks Based on the Voronoi Model

**DOI:** 10.3390/ma14092108

**Published:** 2021-04-21

**Authors:** Guanlin Liu, Youliang Chen, Xi Du, Peng Xiao, Shaoming Liao, Rafig Azzam

**Affiliations:** 1 Department of Civil Engineering, School of Environment and Architecture, University of Shanghai for Science and Technology, Shanghai 200093, China; 181520111@st.usst.edu.cn (G.L.); xi.du@unsw.edu.au (X.D.); 13956284413@163.com (P.X.); 2 Department of Engineering Geology and Hydrogeology, RWTH-Aachen University, 52064 Aachen, Germany; azzam@lih.rwth-aachen.de; 3 School of Civil and Environmental Engineering, The University of New South Wales, Sydney, NSW 2052, Australia; 4 Department of Geotechnical Engineering, Tongji University, 1239 Siping Road, Shanghai 200092, China; engcent@tongji.edu.cn

**Keywords:** rock failure, crack development, fracture mechanism, Voronoi model, energy evolution

## Abstract

The cracking of rock mass under compression is the main factor causing structural failure. Therefore, it is very crucial to establish a rock damage evolution model to investigate the crack development process and reveal the failure and instability mechanism of rock under load. In this study, four different strength types of rock samples from hard to weak were selected, and the Voronoi method was used to perform and analyze uniaxial compression tests and the fracture process. The change characteristics of the number, angle, and length of cracks in the process of rock failure and instability were obtained. Three laws of crack development, damage evolution, and energy evolution were analyzed. The main conclusions are as follows. (1) The rock’s initial damage is mainly caused by tensile cracks, and the rapid growth of shear cracks after exceeding the damage threshold indicates that the rock is about to be a failure. The development of micro-cracks is mainly concentrated on the diagonal of the rock sample and gradually expands to the middle along the two ends of the diagonal. (2) The identification point of failure precursor information in Acoustic Emission (AE) can effectively provide a safety warning for the development of rock fracture. (3) The uniaxial compression damage constitutive equation of the rock sample with the crack length as the parameter is established, which can better reflect the damage evolution characteristics of the rock sample. (4) Tensile crack requires low energy consumption and energy dispersion is not concentrated. The damage is not apparent. Shear cracks are concentrated and consume a large amount of energy, resulting in strong damage and making it easy to form macro-cracks.

## 1. Introduction

Rock fracture has always been one of the most concerning issues in the field of rock engineering practice, such as underground excavation [1,2], rock slope stability [3], seismic influence [4], and enhanced geothermal system [5], etc. The existing mechanical characteristic experiments can only explore the macroscopic failure and cannot directly explore the rock’s compression fracture process. The initiation, accumulation, and development of internal microscopic cracks in the rock are essential for the irreversible damage and brittle fracture of the rock during compression [6,7]. Therefore, the same physical experiment condition is created by numerical simulation to reappear the stress–strain path of the rock material and to study the crack development process, which cannot be observed in the experiment.

There are many ways to explore the evolution of rock cracks using numerical simulation methods, typically classified as continuum methods and discrete methods [8]. The continuum methods mainly focus on the finite element method (FEM), extended finite element method (XFEM), and finite difference method (FDM). The discrete method mainly focuses on the discrete element method (DEM).

In the finite element method (FEM), the local mesh adjustment technique can simulate linear elastic materials [9]. The crack initiation location, angle, and crack growth process were investigated through prefabricated crack experiments [10,11]. However, one of the drawbacks of the FEM is that it cannot capture localization of failure as the lack of an internal length scale, which results in the underlying mathematical problem becoming ill-posed [12]. To avoid such problems, the Extended Finite Element Method (XFEM) was proposed by Belytschko [13]. In XFEM, the finite element mesh and crack are independent of each other, which makes it convenient to analyze discontinuities of cracked bodies, which has led to its widespread application [14,15,16]. XFEM is restricted by the selection of a single crack criterion. The wing cracks appeared in the simulation results, and there were no extended secondary cracks [15,17], so this method is still a gap with the real crack development. With the development of FDM software represented by FLAC, the fast Lagrangian explicit finite difference method is used to solve many complex engineering problems that are difficult to simulate with finite element programs [18,19]. By using the linear elastic fracture mechanics theory and the failure criterion of the stress intensity factor, the fracture development of rock mass with micro-cracks was effectively simulated, and the general characteristics of brittle materials under compression were perfectly captured, including the fracture process and acoustic emission events as well as the stress–strain curve [20,21]. Although the continuum method has made an outstanding contribution to the study of rock fracture mechanisms by many researchers, the mesh of the numerical model is not divisible, and plastic elements are used to represent rock failure. It is also challenging to analyze the discontinuous deformation during fracture, which still has some limitations in reproducing the crack cracking condition. To analyze the interaction of discontinuous deformation, large displacement and rotation in the process of rock failure, the discrete element method (DEM) has attracted more and more attention from scholars [22,23].

The DEM can be conveniently divided into an explicit and implicit formulation, according to the solution algorithm used. Moreover, the popular representatives of explicit DEMs are the Particle Flow Code (PFC) [24] and Universal Discrete Element Code (UDEC) [25]. Unlike other discontinuum codes, PFC can form dense clusters of contact bonded particles to represent rock material, which does not require either a mesh or a complex constitutive model [26,27]. In UDEC, the simulated material is divided into many polygonal discrete blocks with displacement along joints and intact deformation of joint-bounded areas [28,29]. When the stress value between the block interfaces exceeds the tensile or shear threshold, physical discontinuities will occur [30,31]. Rock failure is captured either in terms of plastic yielding of the rock matrix or the discontinuities’ displacements using the polygonal block, as Voronoi tessellation [29,31]. In contrast to conventional discontinuum codes, an advantage offered by PFC and UDEC is that a crack can be modeled as a real discontinuity [28,32,33,34].

A current limitation of PFC is that the ratio of tensile strength to compressive strength is often around 0.25 [27,35,36], which is much higher than the tension–compression ratio of 0.05–0.1 measured in the experiment [37]. Cundall et al. [27] and Cho et al. [35] optimized composite models of ‘cluster logic’ and ‘clump logic’ to solve its defects, so that the tension–compression ratio is controlled within a reasonable range below 0.1. However, these logic models with too many micro-parameter settings make the calibration more complicated and time-consuming, and some parameters lack real physical meaning [27,34,35]. The Voronoi polygonal block model not only restores the connection mode and shape between the blocks, but also has fewer parameters to be set in the model, and the microscopic parameters correspond to the macroscopic physical characteristics, which are more in line with the physical characteristics [34,38,39]. The Voronoi model can control the tensile strength of rock materials by adjusting the tensile strength of the block contact surface. It solves the problem of the ratio of unconfined compressive strength to tensile strength [28,34]. The Voronoi model has the advantage of using simple logical combinations to achieve close to real rock materials.

In this study, the UDEC-Voronoi approach was used to build the numerical models for uniaxial/triaxial compressive and Brazilian split tests, and the microscopic parameters of contacts were obtained for four typical rock samples by calibration and inversion. Then, the uniaxial compressive tests of rock with four different compressive strengths were conducted. The characteristics of changes in the number, angle, and length of cracks in the process of rock failure and instability were monitored. The laws of crack development, damage evolution, and energy evolution were analyzed using the Voronoi model. The failure and instability mechanism of rock material under the discrete block model was discussed. It reveals the failure laws of rock materials at the micro-level and reproduces the whole process of crack evolution under natural conditions.

## 2. Numerical Modeling

### 2.1. Voronoi Model Mechanical Behavior

**Figure 1:**
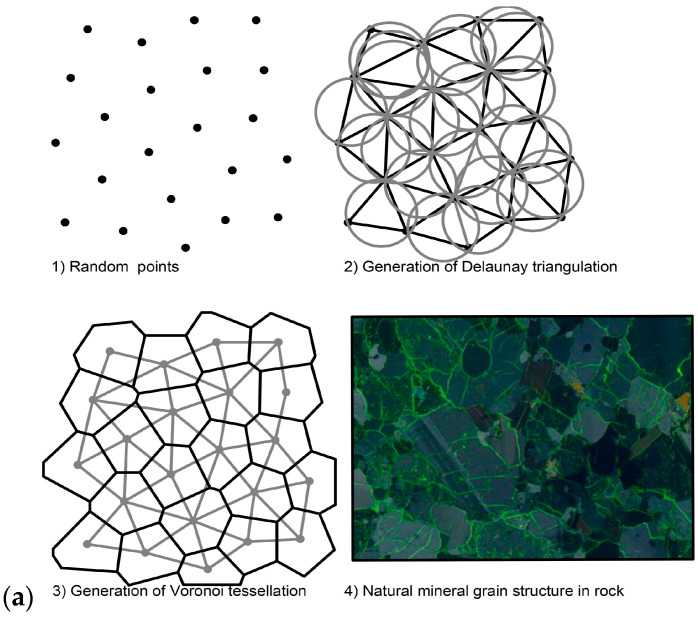

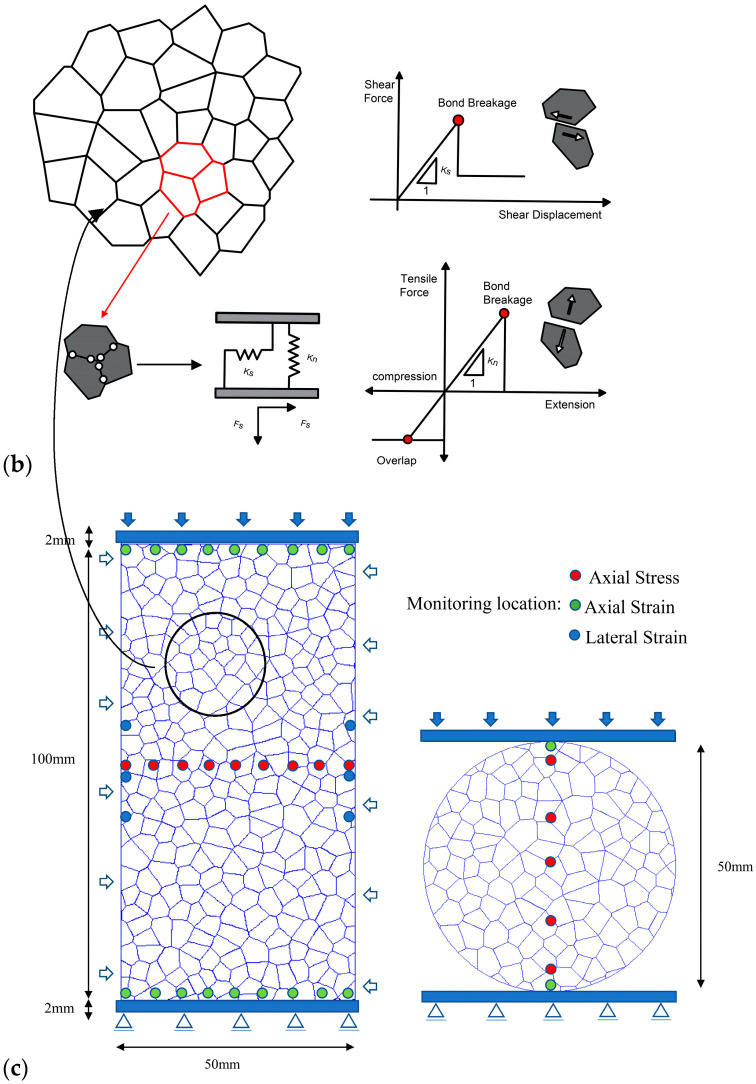
(**a**) Voronoi tessellation generator logic and natural mineral grain structure in rock (modified from [29,43]); (**b**) constitutive behavior of Voronoi model (modified from [44]) Note: The normal force ($F_{n}$) is controlled by the normal stiffness ($k_{n}$), and the shear force ($F_{S}$) is controlled by the shear stiffness ($k_{s}$); (**c**) numerical models of uniaxial/triaxial compressive and Brazilian split tests.

At the microstructure level, the rock material is generally considered to be a combination of many discrete polygonal grains (Figure 1a). The Voronoi tessellation framework has been verified that naturally agrees with the granular meso- or micro-structure of rock materials [29,40,41]. It is mainly composed of the lattice element method and spring network methods [40,41,42]. UDEC has an in-built, automatic generator of the Voronoi tessellation pattern, where a particular region in a model can be subdivided into randomly sized polygons. This assemblage of distinct deformable polygons model is called the Voronoi model. The Voronoi model is made up of a set of lattice nodes that can be regularly and irregularly distributed along the domain [40,41]. Lattice nodes are connected with lattice elements, usually forming a Delaunay triangulation, and each triangle is circumscribed within a circle containing its three vertexes. Finally, the Voronoi polygons are created by constructing perpendicular bisections of all the triangles that share a common side [40,43] (Figure 1a).

The Voronoi polygon blocks are assigned an isotropic elastic deformable material model, which means that failure on the micro-scale only takes place at the block boundaries, not inside them. The mechanical properties of the connection between Voronoi polygon blocks obey the Coulomb friction law. Normal stiffness, shear stiffness, cohesion, friction, and tensile strength can be assigned to the contacts. These properties are referred to as the micro-properties. The Voronoi micro-mechanical behaviors and properties are shown in Figure 1 and Table 1.

In the normal direction, the contact force–displacement relation is assumed to be linear and governed by its normal stiffness $\left(k_{n}\right)$ such that

(1) $$∆\sigma_{n}=-k_{n}∆u_{n}$$

where $∆\sigma_{n}$ is effective normal force increment and $∆u_{n}$ is the normal displacement increment. A limiting tensile strength, $\sigma_{n}^{max}$, is assumed for any contact. If the tensile strength is exceeded$\;(\sigma_{n}\leq -\sigma_{n}^{max})$, then $\sigma_{n}=0$, and the contact is marked as a tensile crack. In the opposite direction, blocks should be compressed and overlap at these contact points and the overlap is controlled by $k_{n}$.

In the shear direction, the response is controlled by contact shear stiffness$\;(k_{s})$. The shear stress ($\tau_{s}$) is limited by a combination of the cohesion$\;\left(C^{cont}\right)$ and friction$\;\left(\varphi^{cont}\right)$. Thus, if

(2) $$\left|\tau_{s}\right|\leq C^{cont}a_{c}+\sigma_{n}tan\varphi^{cont}=\tau_{s}^{max}$$

then,

(3) $$∆\tau_{s}=-k_{s}∆u_{s}^{e}$$

or else, if

(4) $$\left|\tau_{s}\right|\geq \tau_{s}^{max}$$

then

(5) $$\tau_{s}=sign\left(∆u_{s}\right)\;\tau_{s}^{max}$$

where $a_{c}$ is contact areas, $∆u_{s}^{e}$ is the elastic component of the incremental shear displacement and $∆u_{s}$ is the total incremental shear displacement. The contact is marked as a shear crack if Equation (4) is achieved.

The Voronoi model’s time step calculation is based on stiffness, and the connected nodes are generally given higher normal and shear stiffness values to prevent free movement along the joints. The normal stiffness and shear stiffness for the contacts in the numerical model can be derived from the equations in [25]:

(6) $$k_{n}\left(\;k_{s}\right)=10\left[\frac{K+\frac{4}{3}G}{∆z_{min}}\right]\;,\;n=1\sim 10$$

After one contact breaks, forces are redistributed, and it might cause adjacent contacts to break. Tension spring ($k_{n}$) and shear spring ($k_{s}$) work independently of each other. So, when the tension spring fails, the shear spring is still working, and vice versa. It can be further explained as follows: shear and tensile cracks can occur at the same contact at the same time, or only tensile cracks can occur. During the process, there is no need for a complex constitutive model to control cracking behavior. This implies that only contact properties are controlling material response on the macro-scale [34]. Figure 1b illustrates the contact normal and shear behavior.

### 2.2. Modelling Characteristics

According to different compressive strengths, Loc du Bonnet (Ldb) Granite, Augig Granite, Transjuane Sandstone (TS), and Coal, the four typical rock samples from hard rock to weak, were selected as the numerical simulation test samples of this paper. The detailed experimental parameters are shown in Table 2. According to the experimental sample standard recommended by ISRM [45], the numerical models of uniaxial/triaxial compression and Brazilian tension tests were established, as shown in Figure 1c. The specimen’s size for the uniaxial/triaxial compression model is 50 mm × 100 mm (diameter × height), and the diameter of the sample for the Brazilian tension model is 50 mm. The Voronoi block is assigned as elasticity. The connection between the blocks follows the Coulomb slip model with residual strength properties [46]. The setting of the Voronoi block size also influences the failure mode [28,34,43]. Only when the block size is less than 1/10 of the minimum model sample size can the influence of the size be ignored [43,46]. Considering the calculation performance and time consumption, a too-small grain block size is also impractical, making the model calculation time-consuming. Referring to the actual grain size distribution of the selected samples, the average block size of LdB granite and Augig granite samples in the model is 4 mm [28,29,34,47], and the TS and Coal rock samples are 2 mm [28,34,48], which meets the size requirements. The loading rate is set to maintain the displacement rate at 0.01 m/s in both compression and tension tests [34,43]. Triaxial compression can be obtained by applying a constant lateral force to both sides of the uniaxial compression model. The stress and strain of the model during compression can be obtained through the displacement and force monitoring points as shown in Figure 1b. The numerical model for this paper took approximately 28 min to calculate (PC, Inter Core i9-9900k 3.6 GHz, Win10 64 bit). The model is in equilibrium and the calculation converges when the real-time maximum unbalance force is reduced to 1% of the initial maximum unbalance force.

## 3. Microscopic Mechanical Properties of Voronoi Model

In the Voronoi model, using the UDEC built-in FISH program to track and monitor the changes of the connection properties between blocks, the fracture evolution process of micro-cracks can be observed effectively. However, it is necessary to verify the validity of the model before numerical simulation. Therefore, we need to conduct a sensitivity analysis on the microscopic parameters of the model to prove that the microscopic mechanical properties of the numerical model can match the macroscopic properties of the rock. The micro-properties to be calibrated in the model are shown in Table 2. Previous studies [34,43,44] have analyzed the parameters of Voronoi to some extent. In this section, the classical LdB granite sample was used to conduct supplementary studies on the sensitivity of microscopic parameters by means of controlling variables, and feasible correction suggestions were proposed. Limited to space, only the representative simulation results are listed below.

### 3.1. Micro-Properties Sensitivity Analysis

#### 3.1.1. Micro Deformability Properties

- Young’s modulus of micro-block ($E_{b}$)

**Figure 2:**
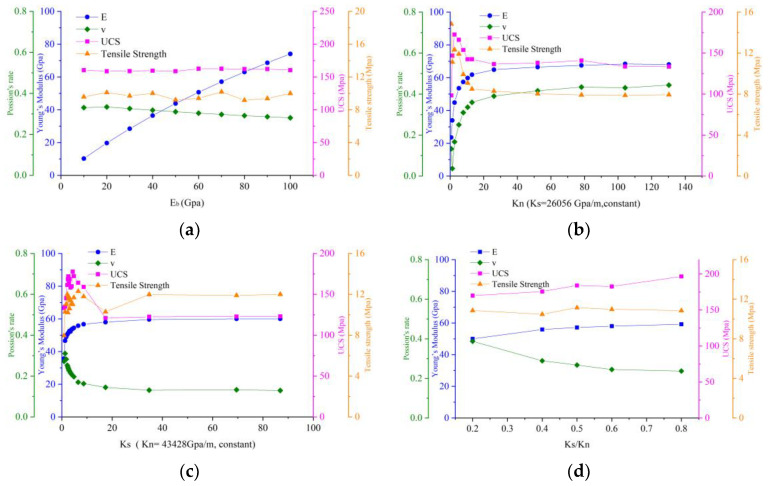

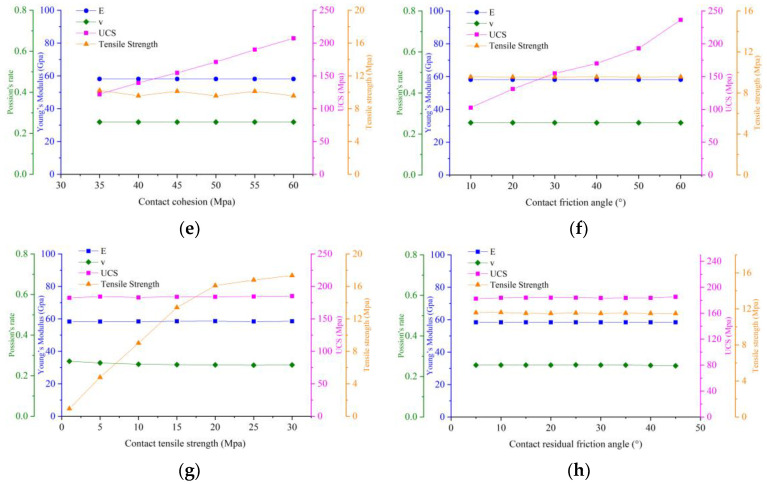
The mechanical properties of the macroscopic sample according to each influencing micro-connection parameters: (**a**) Young’s modulus of the block $\left(E_{b}\right)$, (**b**) normal stiffness ($k_{n})$, (**c**) shear stiffness ($k_{s})$, (**d**) stiffness ratio (ks/kn), (**e**) contact cohesion ($c_{j})$, (**f**) contact friction angle ($\varphi_{j})$, (**g**) contact tensile strength ($\sigma_{tj})$, (**h**) contact the residual friction angle ($\varphi_{jr}$).

From Figure 2a, Young’s modulus of the micro-block $E_{b}\;$plays a leading role in Young’s modulus *E* of the entire rock and the relationship between the two is increasing linearly, while $E_{b}$ will cause a slight decrease in Poisson’s ratio, and has little effect on the strength. When the value of $E_{b}$ gradually increases, the polygons behave more stiffly. The cumulative deformation mainly focuses on contacts and ignores the elastic deformation of the block. Thus, the material’s Young’s modulus increases and Poisson’s ratio decreases. When $E_{b}$ is assigned a lower value, the polygons are softer and the cumulative deformation mainly focuses on blocks, and thus the material’s Young’s modulus decreases and Poisson’s ratio decreases. It is also shown that $E_{b}$ has a negligible influence on strength.

- Contact normal stiffness ($k_{n}$) and shear stiffness ($k_{s}$)

It can be seen from Figure 2 that the Young’s modulus *E* of the rock is also affected by the $k_{n}$ and $k_{s}$ between the microscopic joints. The increase in $k_{n}$ and *k*_s_ in the early stage will cause *E* to increase exponentially, which far exceeds the influence of $E_{b}\;$on *E*. However, for the Poisson’s ratio *v* of the material, $k_{n}$ becomes an exponentially positive correlation and $k_{s}$ becomes an exponentially negative correlation. The Young’s modulus (*E*) and Poisson’s ratio (*v*) of the rock are mainly affected by $k_{n}$ and $k_{s}$ in the stiffness ratio between 0.1 and 1.1 (*k_n_*/*k_s_* is close to 0.9–10). When this ratio is exceeded, both *E* and *v* of the rock will tend to a constant value.

- Contact stiffness ratio (*k_s_*/*k_n_*)

Some scholars have verified that the ratio of shear stiffness to normal stiffness determines Poisson’s ratio of macro samples [28,34]. However, different stiffness ratios have different effects on the material’s Poisson ratio. The smaller stiffness ratio will increase the elasticity of the rock, resulting in a larger Poisson’s ratio. If the stiffness ratio taken is too small, the axial stress is much greater than the vertical stress. It will result in an internal force imbalance that cannot be loaded statically.

#### 3.1.2. Micro Strength Properties

- Contact-Cohesion ($c_{j}$) and Contact-Friction angle ($\varphi_{j}$)

In Figure 2, the contact-cohesion and contact-friction angles do not affect the elastic modes, Poisson’s ratio, and tensile strength, which only have the linear incremental relationship of UCS. It is shown that contact-cohesion and contact-friction angle are the main factors affecting strength. To further explore the influence of contact-cohesion and contact-friction angle on the strength of intact rock, triaxial compression simulations are established. The confining pressure range is suggested in $0\leq \sigma_{3}\leq \mathrm{UCS}/10$ [50]. Therefore, the confining pressure value of this experiment is 0, 4, 8, 12 Mpa, respectively. The contact-cohesion is assumed to be from 35 Mpa to 60 Mpa and increments at 5 Mpa intervals while keeping the other parameters constant. Variation of the contact-friction angle are between 10° and 60° with increments of 10°. The friction angle (φ) and cohesion (c) of intact rock were obtained [27,44] using $\varphi =sin^{-1}\left(\frac{N_{\varphi }-1}{N_{\varphi }+1}\right)$, and $c=\frac{\sigma_{c}}{2\sqrt{N_{\varphi }}}$. $N_{\varphi }$ was defined [27] by the peak strength ($\sigma_{f}$) and for confinements in the range $P_{0}\;$(0 Mpa) − $\;P_{1}$(12 Mpa) via $N_{\varphi }=\frac{\sigma_{f}\left(P_{1}\right)-\sigma_{f}\left(P_{0}\right)}{P_{1}-P_{0}}$.

**Figure 3:**
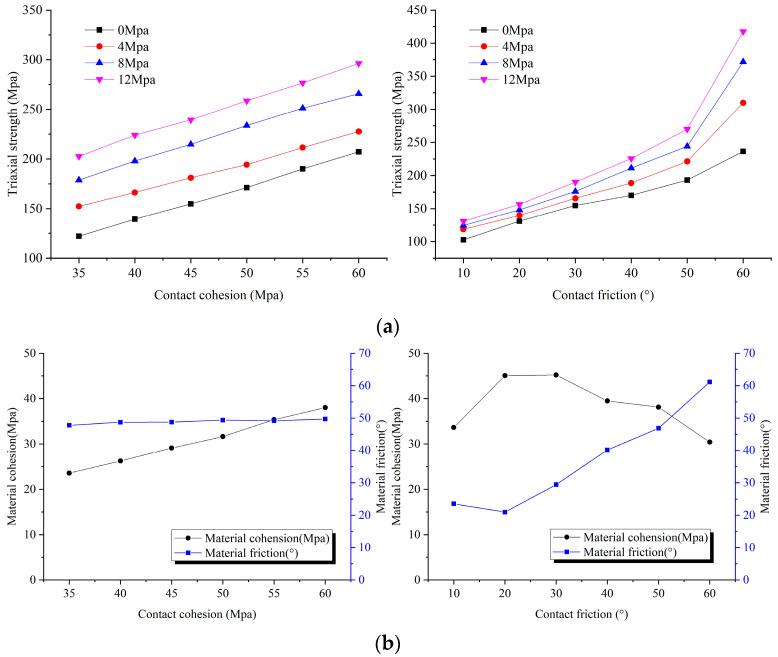
Effect of contact cohesion ($c_{j})$ and contact friction angle ($\varphi_{j})$ on (**a**) the material triaxial compressive strength; (**b**) the material friction angle and cohesion.

In Figure 3, the contact-cohesion is only linearly positively related to the cohesion of intact rock. The contact-friction angle not only plays a major role in the internal friction angle of the material but also has a greater influence on the cohesion of intact rock, especially when the contact-friction angle is larger. The explanation may be that the Voronoi model is a convex polygon block, and the friction angle between the blocks is too large, which will cause the interlocking effect to be formed, and also make the strength of the sample unrealistically large. Therefore, it is not desirable to take too large a value of the internal friction angle.

- Contact-Tensile strength ($\sigma_{tj}$)

In the Brazilian tension test, the indirect tensile strength of a cylindrical sample is given by $\sigma_{t}=\frac{P_{max}}{\pi Rt}$. where $P_{max}$ is the maximum load at failure, *R* is the radius of the sample, and *t* is the thickness of the sample. In Figure 2g, the tensile strength of intact rock is only controlled by the micro tensile strength. Therefore, the Brazilian tension numerical model can be established to adjust the micro tensile parameters so that the tensile strength of the model reaches the experimental tensile strength.

#### 3.1.3. Post-Peak Stress Parameters

When the peak strength exceeds, the connection bond between blocks will be broken to generate cracks. Currently, there is no residual cohesion ($c_{jr}$) and residual tensile strength ($\sigma_{tjr})$ in cracks, so the value is 0. The residual friction angle ($\varphi_{jr}$) is an inherent property of the polygon block and still exists. In Figure 2f, the residual friction angle does not affect the deformation and strength before peak strength. The residual friction angle mainly plays a certain carrying role in the post-peak damage [51]. The carrying capacity is mainly provided by generating greater frictional resistance for the micro-block’s dislocation motion. When the residual friction angle value is too large, it will also cause the rock sample to appear interlocked, which is a phenomenon and result of the post-peak enhancement effect. To meet the actual rock sample damage pressure, by trial and error, the value should not be greater than half of the friction angle.

### 3.2. Calibration Procedure and Results Analysis

The elastic parameters ($E_{b}$,$v_{b}$,$k_{n}$,$k_{s}$) and the strength parameters ($c_{j}$,$\varphi_{j}$,$\sigma_{tj}$) of microscopic blocks control the deformation (Ε,v) and strength (c,φ,$\sigma_{t}$) of intact rocks. The initial parameters of the model were set according to the experimental data (i.e., $E=E_{b}$,$v=v_{b}$). The block size was chosen according to mineralogy. The contact stiffness ratio (*k*_s_/*k*_n_) is determined to macro-Poisson’s ratio. Once the contact stiffness ratio was set, both the normal stiffness ($k_{n}$) and block deformability ($E_{b}$) were altered to fit the macro-Young’s Modulus (E). The calibration procedure of the numerical model followed the procedure outlines by Christianson et al. [52], Kazerani and Zhao [34], and Gao and Stead [28]. By trial and error, a unique set of contact parameters, which satisfies the material properties, including Young’s model, Poisson’s ratio, tensile strength, friction angle, cohesion, and uniaxial compressive strength, were established (see Table 3).

**Figure 4:**
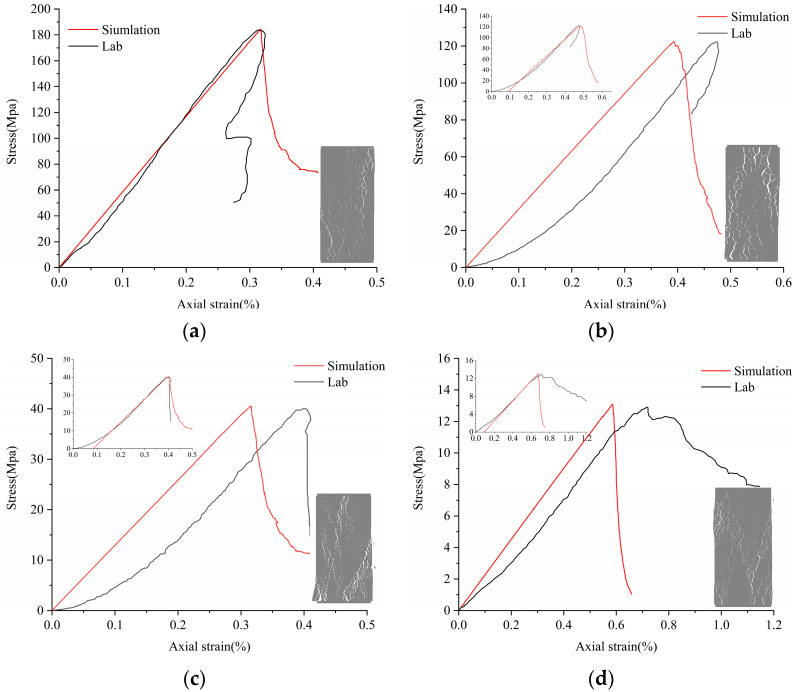
Calibration of Voronoi models to (**a**) LdB granite, (**b**) Augig granite, (**c**) TS, and (**d**) coal.

Original cracks and pores exist in real rock samples, so the stress–strain curve in the experimental data has a crack closure phase in the early stages, which is not considered in the Voronoi model. When the rock sample is compressed, the stress–strain curve jumps over the crack closure and directly goes through the elastic, elastic–plastic, and plastic yield. Therefore, it is necessary to remove the nonlinear phase (crack fracturing stage) in the early stage of the experiment when correcting the model. The stress–strain curve of the modified model was moved to the right to the elastic part of the experimental stress–strain curve (the thumbnail in the upper left corner of the model), and the stress–strain curve of the corrected model is consistent with the experimental results, as shown in Figure 4. Due to the high degree of denseness of LdB rock samples, there are very few original cracks and pores, and the pre-pressing stage is not obvious. Therefore, the LdB rock numerical model can ignore the pre-nonlinear stage and correct the model directly. The error values of macro parameters obtained by the four simulation samples are all less than ±7%, which is close to the actual results (see Table 4). The tensile compression ratio is mainly between 0.05 and 0.08, which is consistent with the experimental results. Based on the above conclusions, the microscopic calibration parameters can accurately reflect the macroscopic mechanical properties of rock samples.

## 4. Micro-Crack Evolution in the Rock Fracture Process

Uniaxial compression experiments are extensively used to study the failure process of intact rock. Meanwhile, rock is a brittle material, and its failure process cannot be obtained by direct observation. To understand the evolution of fracture development, it is necessary to reconstruct the fracture development process by numerical simulation. According to the failure criterion of the Voronoi model principle, the contact surface between the two blocks could be monitored and judged. If the tensile strength/shear stress is exceeded, the contact will be recorded as a tensile crack/shear crack.

### 4.1. Strain–Stress Crack Analysis

**Figure 5:**
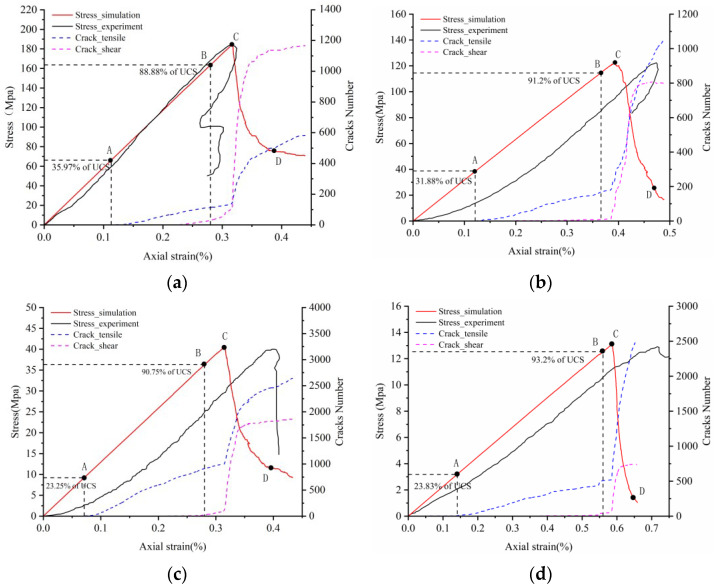
Comparing the experimental and simulated stress–strain and crack–strain curves for various rock specimens: (**a**) LdB granite, (**b**) Augig granite, (**c**) TS, and (**d**) coal. Note: Initial crack threshold (Point A), crack damage threshold (Point B), peak failure (Point C) and post-peak residual (Point D).

Eberhardt et al. [53] conducted a series of unconfined compressive tests on brittle rock and found that there are two main thresholds before the peak strength: the crack initiation threshold ($\sigma_{ci}$) and the crack damage threshold ($\sigma_{cd}$). Both were identified through acoustic emission and strain monitoring. The crack initiation threshold is determined by the first appearance of the crack and the beginning of stable growth. The crack damage threshold is mainly determined by the unstable development of cracks, which is mainly manifested as the cracks begin to increase sharply. Both the crack initiation and damage thresholds were successfully captured in the Voronoi models. Cai and Hoek et al. [54,55] obtained the distribution range of the crack initiation threshold and crack damage threshold in the range of 30–60% and 70–90% of uniaxial compressive strength. Xue et al. [56], through the data statistics of the uniaxial compression experiment, found that the crack damage threshold of most sedimentary rocks is between 60–90%. In Figure 5, the simulation result shows that the crack initiation thresholds (Point A) of the four rock samples under the uniaxial compression model were 35.97%, 31.88%, 23.25%, and 23.83% of UCS, respectively. These values are slightly lower than 30–60% of the experimental statistical value of UCS, which is mainly due to the fact that the compaction stage in the early stage is not considered, which makes the initial threshold of cracks move forward. The crack damage threshold values (Point B) of four rock samples were 88.88%, 91.2%, 90.75% and 93.2% of UCS, respectively and the values are in line with the experimental statistics range.

Hoek and Bienawski [57] divided crack development into five stages: consolidation, elastic deformation, stable crack development, unstable crack development, and post-peak stage. Because the Voronoi model did not consider the original cracks or pores, there will be no crack closure in the simulated stress–strain curve. In this model, the development of cracks mainly includes the elastic deformation stage (0–A), stable crack development (A–B), unstable crack development (B–C), and post-peak stage (C–D); see Figure 5.

At the elastic deformation stage, the four rock samples are mainly deformed by pressure and do not produce cracks. From the A–B stage, cracks gradually began to germinate. This period is the initial development stage of crack and mainly focuses on tension cracks. A small number of shear cracks begin to initiate at this stage. When the B–C stage is reached, the damage of the rock sample develops rapidly. The growth rates of tension crack and shear crack increase. When the crack development reaches the peak strength (point C), the rock sample still keeps stability and has an effective bearing capacity. After exceeding the peak strength point (C–D stage), the shear crack and tensile crack increase sharply and lose the bearing capacity in a short time.

Comparing the numerical simulation results of the four rock samples, the number and increment of shear cracks in hard rock are significantly higher than those of tensile cracks, while the number and increment of tensile cracks in the weak rock are higher than those in the shear crack. It shows that shear cracks are the main form of hard rock failure, while tensile cracks are the main form of failure in weak rock. No matter the kinds of rock fractures, the generation, and evolution of shear cracks all occur near the peak strength. When the peak strength is exceeded, the number of shear cracks decreases rapidly and tends to a lower value. The development of the tensile crack runs through the whole failure process.

### 4.2. Crack Evolution Process and Crack Angle Analysis

**Figure 6:**
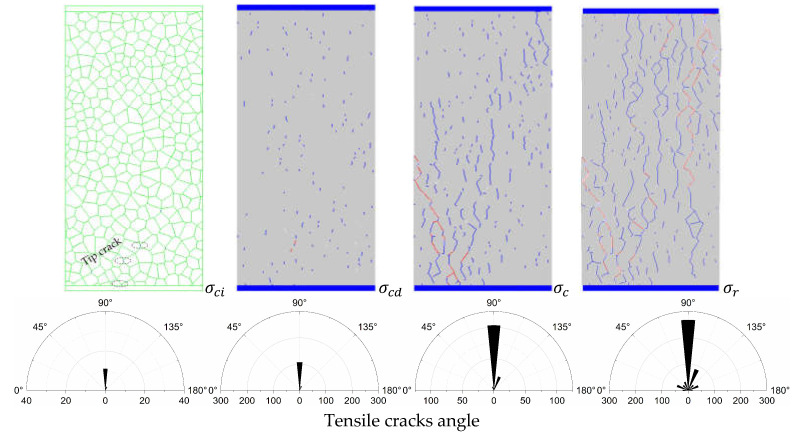

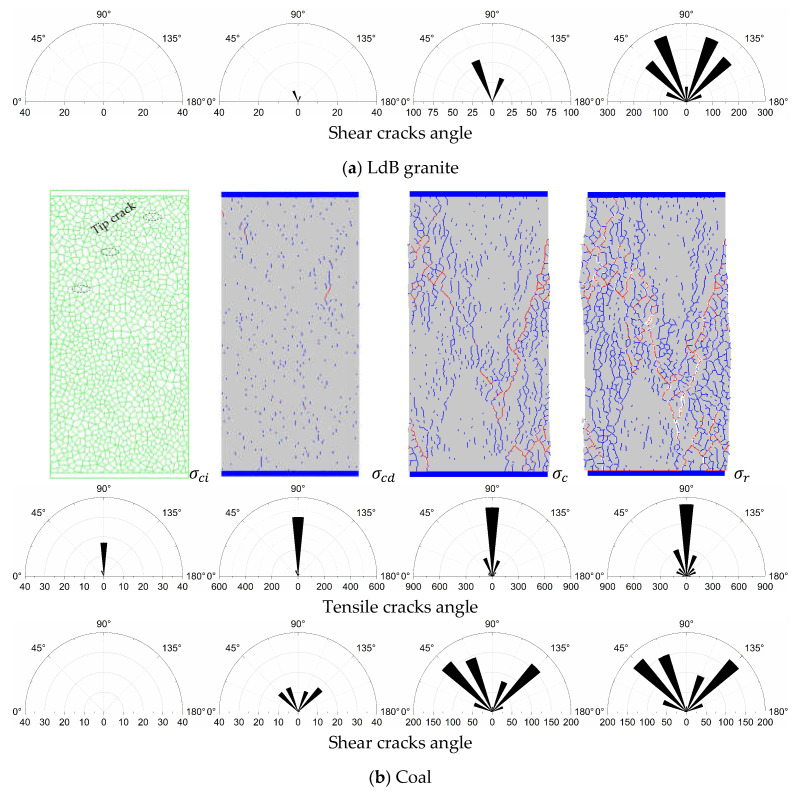
Crack evolution and crack angle statistics during hard rock and weak rock fracture: (**a**) LdB granite (**b**) Coal. Note: The blue line represents the tensile crack and the red line represents the shear crack.

To better display the fracture trend, the whole process of crack evolution is quantitatively described by selecting rock samples at the initial crack threshold point ($\sigma_{ci}$), crack damage threshold point ($\sigma_{cd}$), peak failure point ($\sigma_{c}$)and post-peak residual point ($\sigma_{r}$), as shown in Figure 6. Due to limited space, only LdB granite and coal samples with the highest uniaxial compression strength (184.5 Mpa) and the lowest (13.1 Mpa) of the four samples were selected to characterize hard rock and weak rock. It can be seen from the crack evolution process of LdB granite and coal shown in Figure 6.

When the stress reaches the initial crack threshold ($\sigma_{ci}$), the damage distribution of the cracks is discrete, and mainly tensile cracks. The cracking angle is mostly concentrated at 90°, the cracks are small, and the distribution density is low, in which no shear cracks are generated.

As the load increases and reaches the crack damage threshold ($\sigma_{cd}$), the density of the tensile crack distribution gradually increases, and the developed cracks mainly occur at the tip of the polygonal block and grow steadily in the form of random distribution. At the same time, the shear cracks began to appear, and the cracking angles were mainly 45° and 70° (135° and 150°), respectively, and the tendency of crack accumulation began to appear in some areas of the numerical model.

When the peak failure point ($\sigma_{c}$) is reached, the distribution densities of tensile cracks and shear cracks increase significantly, and the density of the tensile cracks’ distribution is higher than that of the shear cracks. Tension cracks and shear cracks inside the model began to accumulate based on the original cracks. The microcracks gradually formed fissures and further expanded into multiple fissures. The development of the fissures was mainly concentrated on the diagonal line of the rock sample. After the fissures formed from the diagonal corners, they gradually expanded to the center of the rock sample along both ends of the diagonal line.

When loaded to the post-peak residual point ($\sigma_{r}$), the tensile cracks mainly extend along the loading direction of 90°, and the shear cracks mainly extend along the direction of 45–70° (135–150°). The distribution density and distribution area of the cracks increase significantly and finally form many macroscopic penetrating fractures.

Whether it is hard rock or weak rock in uniaxial compression failure, the tensile failure caused by the accumulation of tensile cracks mainly occurs along the direction of loading (failure occurs in the direction of 90°), and the shear failure formed by the shear cracks mainly occurs in the directions of 45° and 70° (135° and 150°), which is consistent with the macroscopic fracture formed in the experiment.

### 4.3. AE Event Count

The cracks inside the rock material release elastic waves when they are generated and extended. The way to count the number of elastic waves sent out by cracks is called acoustic emission (AE) count event. For the Voronoi model, the AE event can be simulated by counting the number of fracture joints. Although the numerical simulation method cannot completely reproduce the same situation as the experiment, the statistical contact faults can also better reproduce the number of cracks changes in the rock to characterize the acoustic emission. This method of AE simulation has been well applied in previous studies [58,59,60].

**Figure 7:**
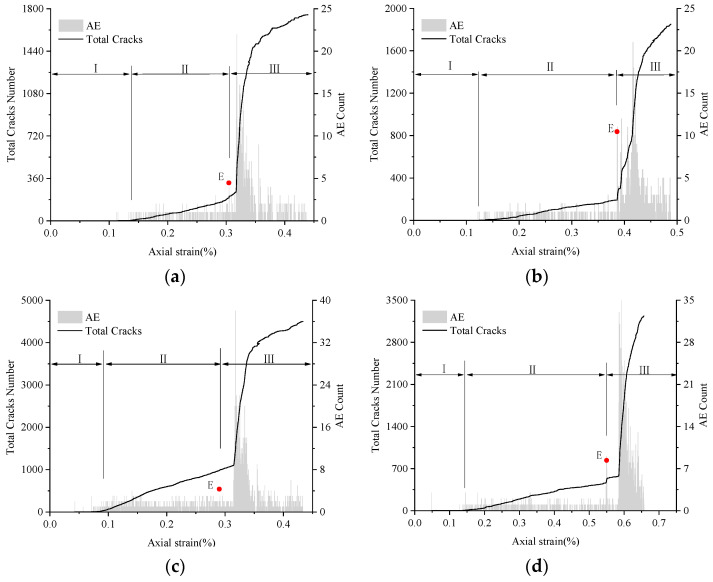
Total cracks and AE curves of various rock specimens. (**a**) LdB granite, (**b**) Augig granite, (**c**) TS, and (**d**) coal. Note: Red point E represents the information recognition point of the rock failure precursor.

As can be seen from Figure 7, the four rock samples’ acoustic emission characteristics are roughly divided into three stages: the initial silence period, the intermediate stabilization period, and the last peak period.

Initial silence period (segment I): This period corresponds to the line elasticity phase of stress–strain, which is mainly the elastic deformation behavior that occurs in the pressure between blocks, so the acoustic emission signal is very weak or not even in this segment. The small amount of AE event that appears in TS sand and coal in this period is caused by uneven force due to the uneven block shape.

Intermediate stabilization period (segment II): After the accumulation of energy during the line elastic phase, micro-cracks develop gradually inside the rock, and the AE event begins. As the loading increases, the number of AE events begins to increase steadily; this phase takes a long time.

Last peak period (segment III): When loading into the line elasticity to yield the weakening phase is excessive, the number of cracks increase and the AE event count begins to increase dramatically. At this time, before the peak will appear a convex peak value. When the pressure load exceeds the peak strength, the micro-crack begins to expand, evolve and gradually develop into a penetrating macro-crack. The AE event count drops sharply after reaching its maximum peak, at which point the rock sample has broken. The beginning of the last peak stage indicates that the rock has entered the yield failure stage, so the higher peak value of the bulge can be used as the information recognition point of the rock failure precursor (in Figure 7, Point E).

These three stages of AE counts have good consistency with the experimental test [53,61], which further proves that the Voronoi model is effective in simulating the rock fracture process. Combined with Figure 5 and Figure 7, it can be seen that the process of hard rock and weak rock crack generation, propagation and penetration into the macro-crack is very short. The acoustic emission signal is strong and concentrated, showing the brittle failure characteristics of the rock. Using the obvious characteristics of acoustic emission count segmentation, recording the failure precursor information recognition point can also effectively provide a safety warning for rock fracture.

### 4.4. Crack Damage Analysis

**Figure 8:**
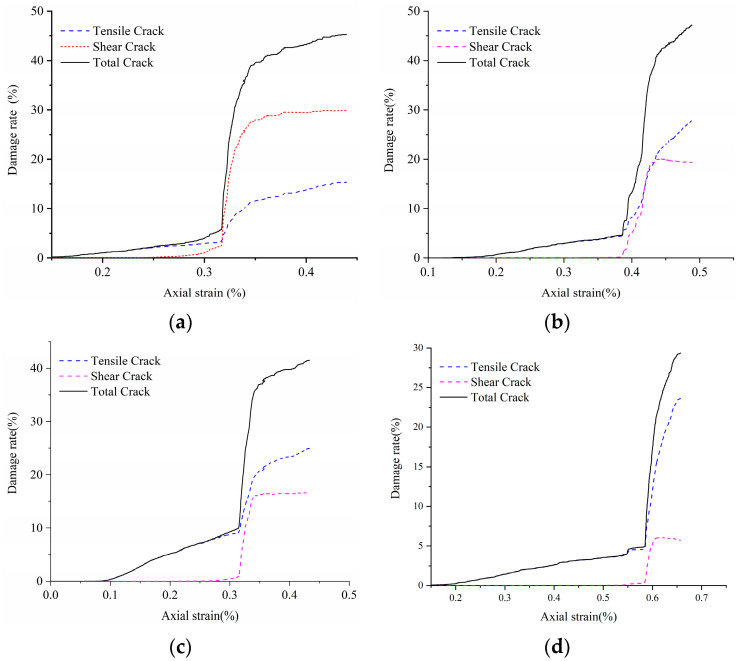
Damage rate of various rock specimens. (**a**) LdB granite, (**b**) Augig granite, (**c**) TS, and (**d**) coal.

On the microscale, the formation of fractures is the result of microscopic crack generation and accumulation. Based on the theory of damage mechanics, the damage degree of intact rock can be defined as $L_{crack}=\frac{L_{D}}{L_{O}}\times 100\%$; where $L_{D}$ is the total length of cracks that have broken at different times, $L_{O}$ is the total length of contacts without damage, and $L_{crack}$ is the damage degree of intact rock in crack length. In Figure 8, when the crack damage value reaches 6–10%, the rock is close to the peak strength. When the damage degree exceeds 10%, the shear crack damage value increases greatly, all the rock samples reach the peak strength and damage occurs. The rock gradually loses its effective bearing capacity. The large increase of shear crack length is an important signal of rock failure. When the crack damage rate of rock is more than 45%, most of the rock has lost its bearing capacity and all of them are destroyed.

Based on the above analysis of crack damage rate, we can further calculate the damage model of the rock rupture process. The scholars Kachanov et al. [62] defined the damage variable as $D=\frac{A_{d}}{A}$. where *D* is the cumulative damage variable, $\;A_{d}$ is the area of the material damage cross-sectional at a certain period, and *A* is the cross-sectional material area without initial damage.

Suppose that $C_{0}$ is the total cumulative crack length of the rock sample from initial no damage to complete loss of bearing capacity. The cumulative crack length per unit area damage is $C_{w}$,

$C_{w}=\frac{C_{0}}{A}$. where the cross-sectional area of damage reaches is $A_{d}$, and the cumulative crack length is $C_{d}$,

$C_{d}=C_{w}A_{d}=\frac{C_{0}}{A}A_{d}$.

Thus, the damage variable can be converted to $D=\frac{C_{d}}{C_{0}}$.

It is difficult for rock samples to achieve an absolute complete failure mode during compression. Therefore, the total crack length of rock samples under axial residual stress is regarded as the cumulative total crack length of rock samples at the complete failure time. From the previous analysis, it can be seen that the rock will completely lose its bearing capacity when the crack damage rate exceeds 45%. Therefore, the crack length with the crack damage rate of 50% is used as the total cumulative crack length.

Based on the crack length and the strain equivalence principle [63], a damage constitutive model of hard to weak rock specimens under uniaxial compression is established:

(7) $$\sigma =E\varepsilon \left(1-D\right)=E\varepsilon \left(1-\frac{C_{d}}{C_{0}}\right)$$

**Figure 9:**
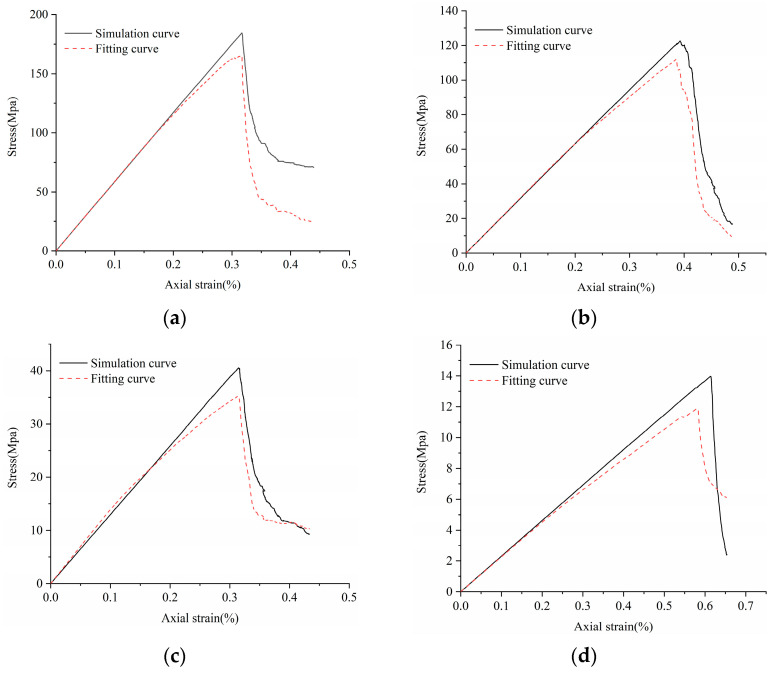
Uniaxial compression damage constitutive equation fitting curve and numerical simulation curve of various rock specimens. (**a**) LdB granite, (**b**) Augig granite, (**c**) TS, and (**d**) coal.

Figure 9 shows the stress–strain curve fitted by the damage constitutive equation of rock samples based on crack length. The fitting curve is in agreement with the numerical simulation curve. In summary, it is feasible to use the crack length parameter to reflect the damage evolution characteristics of rock samples.

### 4.5. Characteristics of Macroscopic Failure

**Figure 10:**
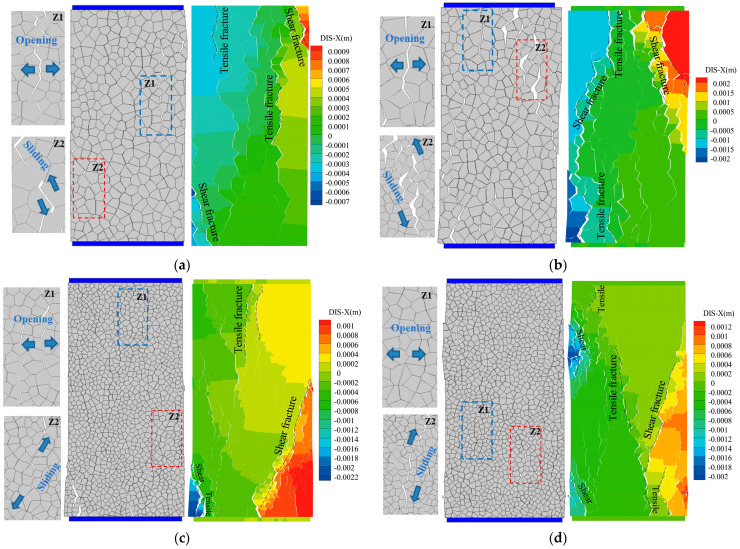
Macroscopic fracture propagation and Horizontal displacement contour of various rock specimens. (**a**) LdB granite, (**b**) Augig granite, (**c**) TS, and (**d**) coal. Note: Insets Z1 and Z2 are zoomed-in plots.

With the increasing load, the microscopic tension and shear cracks in the rock accumulate continuously and eventually form macro-fractures. According to the trend of the fracture and the relative movement directions of the blocks on both sides, it can be divided into the tensile fracture and shear fracture. If the fracture angle was vertical or sub-vertical (i.e., parallel to the loading direction) and the blocks on both sides of the crack move in the opposite direction (i.e., opening), it is defined as a tensile fracture. If the fracture angle was oblique and the blocks on both sides of the crack are sliding, it is defined as a shear fracture. Combined with the horizontal displacement contour plot, the macroscopic failure trend curves of tensile fracture and shear fracture can be obtained, as shown in Figure 10.

### 4.6. Energy Evolution Pattern

The principle of an AE event is mainly to record the energy information changes in a certain frequency band, but it cannot accurately detect the fracture information of weak joints. In the Voronoi model, the fracture between the contact surfaces of blocks will release the strain energy. The energy evolution characteristics of each crack can be obtained by recording the dissipation information of strain energy when cracks are generated. For each contact, the energy accumulation is governed using Equations (8) and (9):

(8) $$U_{jct}=-\frac{1}{2}\left(f_{n}+{f_{n}}^{\prime }\right)u_{n}$$

(9) $$U_{jcs}=\frac{1}{2}\left(f_{s}+{f_{s}}^{\prime }\right)u_{s}$$

where $U_{jct}$/$U_{jcs}$ is the energy accumulation in tension/shear crack, $f_{n}/f_{s}$ and ${f_{n}}^{\prime }/{f_{s}}^{\prime }$ are the normal/shear stress of contact for current and previous time steps, respectively, and $u_{n}/u_{s}$ is the normal/shear displacement of contact for the current time step.

**Figure 11:**
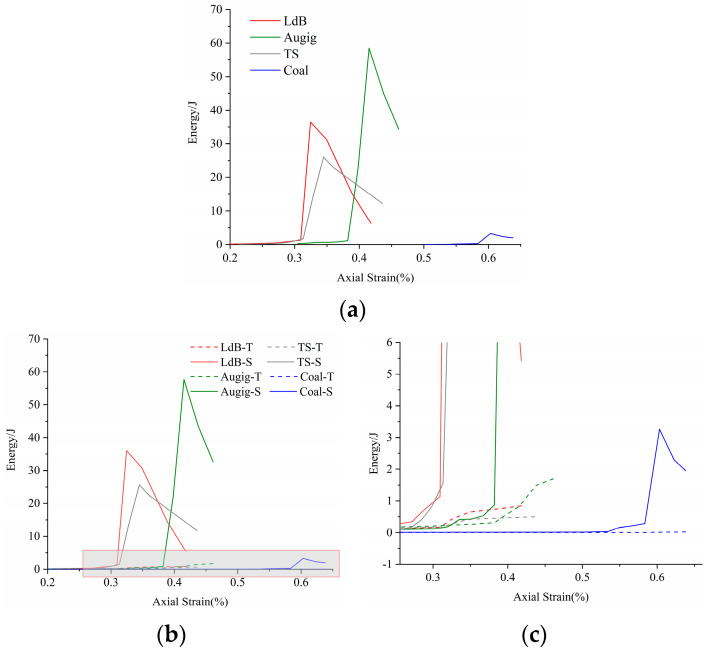
Energy accumulation and dissipation of four rock samples. (**a**) Energy change curve; (**b**) tensile crack(-T) and shear crack(-S) energy change curve; (**c**) locally enlarged view for tensile crack energy change curve.

At the macro-scale, Figure 11a shows the whole process of energy accumulation and dissipation of four rock samples from hard rock to weak rock. Hard rock gathers more energy before failure than weak rock does, but the energy dissipates faster after failure than weak rock.

**Figure 12:**
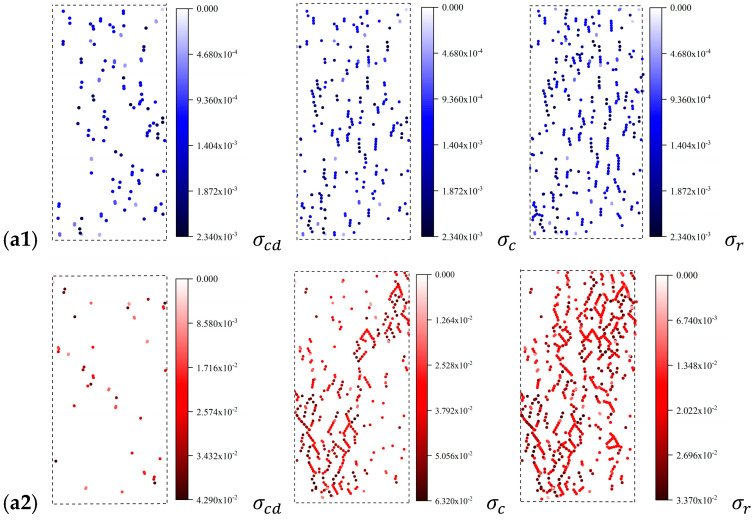

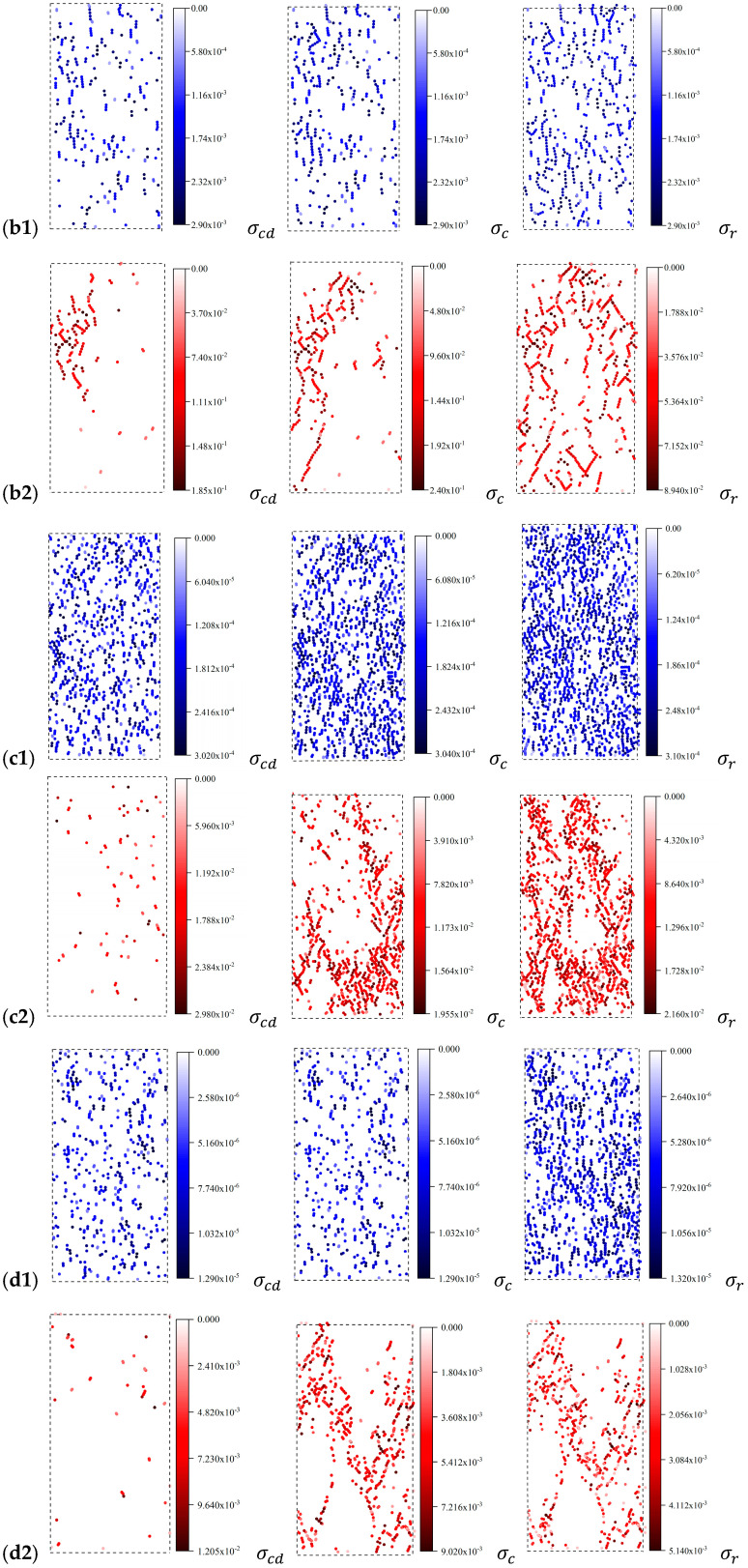
Spatial distribution of the dissipated energy in four rock samples: (**a**) LdB granite, (**b**) Augig granite, (**c**) TS, and (**d**) coal. Note: (1) **a1**, **b1**, **c1** and **d1** are the dissipated energy of tensile cracks, and **a2**, **b2**, **c2**, and **d2** are the dissipated energy of shear cracks; (2) the color of the spheres represents the energy dissipated by cracks.

At the micro-scale, see Figure 11b or Figure 12. Although the energy of tensile cracks keeps gathering, it is far less than that of shear cracks. The changing trend of total energy dissipation is consistent with that of shear cracks. The energy change caused by shear cracks is the main control factor of total energy dissipation. When the tensile crack is broken, the required energy is small and the energy is not concentrated. The damage caused by tensile cracks is not obvious. However, in the energy evolution process of shear cracks, energy dissipation is concentrated, and the macroscopic fractures formed are apparent. This indicates that the shear cracks cause strong damage to the rock.

## 5. Discussion and Conclusions

This paper uses the Voronoi discrete block model to reproduce the whole process of the crack rupture effectively. The microscopic connection parameters between the blocks are the basis for the establishment of the Voronoi model. Many numerical simulations were used to analyze the sensitivity of the microscopic parameters. Therefore, the relationship between the microscopic block’s mechanical properties and the macroscopic sample was obtained through sensitivity analysis. Then, the micro-parameters of the material were inverted through the stress–strain curve. Finally, the process of calibrating the model was quantified, and a reliable numerical model was obtained. If the rock sample has a high degree of compactness, the numerical model can ignore the previous non-linear stage and directly correct it.

(1) The crack development process of the four simulated samples is consistent with the experimental results, and all have experienced elastic deformation, stable crack development, unstable crack development, and post-peak failure. The period between the damage threshold and the peak strength is the most critical period for crack development. Before reaching the damage threshold, it is mainly the initiation and stable development of tensile cracks and it is accompanied by a few shear cracks. The appearance of tensile cracks earlier than shear cracks proves that the tensile resistance of rocks is much weaker than the shear resistance, and that the initiation of internal defects is mainly caused by tensile failure. When the damage threshold is exceeded and close to the peak strength, the internal damage rises sharply and develops into tensile–shear mixed damage. Although the macroscopic failure displacement is not obvious, a large amount of damage occurs inside the rock, reaching the limit of bearing capacity.

(2) Tensile cracks first appeared and mainly developed in the tip part of rock blocks, with a cracking angle of 90°. Tensile cracks formed along the loading direction and developed throughout the whole fracture process. The development of shear cracks is mainly concentrated near the peak strength, with 45° and 70° (135° and 150°) as the main cracking angle, which is the main indicator of the rock damage threshold. The damage caused by shear cracks is the main form in hard rock, while tensile cracks are the main form of damage in weak rock. However, no matter what kind of rock is damaged, the process of crack generation and expansion until it becomes a macro fracture is very short, showing the characteristics of brittle fracture of the rock. The development of micro-cracks is mainly concentrated on the diagonal of the rock sample and gradually expands to the middle along the two ends of the diagonal. Finally, the penetrating fractures along the vertical and horizontal dip angles are formed.

(3) The acoustic emission characteristics of the numerical model can be roughly divided into three stages: the initial silent period, the intermediate stable period, and the last peak period, which is consistent with the acoustic emission characteristics of the experimental statistics and can effectively capture the crack changes. The maximum number of acoustic emission (AE) events appears slightly earlier than the peak strength, and the identification point of failure precursor information can effectively provide a safety warning for the development of rock fracture.

(4) When the crack length damage rate reaches 10%, the rock reaches its peak strength, and when it exceeds 45%, the rock is destroyed. The uniaxial compression damage constitutive equation of the rock sample with the crack length as the parameter is established, which can better reflect the damage evolution characteristics of the rock sample.

(5) In the process of rock rupture, tensile failure requires low energy consumption, the energy dispersion is not concentrated, and the damage is not obvious; while the derived energy of shear cracks is concentrated and destructive, macroscopic cracks are easily formed.

## Figures and Tables

**Table 1:** Voronoi model micro-properties [25].

Young’s Modulus	$E_{b}$	Voronoi Block Elastic Properties
Poisson’s Ratio	$v_{b}$	
Normal Stiffness	$k_{n}$	Voronoi contact elastic properties
Shear Stiffness	$k_{s}$	
Cohesion *	$c_{j}$	Voronoi contact strength properties
Friction Angle *	$\varphi_{j}$	
Tensile Strength *	$\sigma_{tj}$	

* both peak and residual properties.

**Table 2:** Rock macro-properties (experiment data).

		Hard Rock ⟶ Weak Rock			
Lithology		Loc du Bonnet [23] Granite	Augig [34,47] Granite	Transjuane [34,48] Sandstone	Coal [28,49]
UCS (Mpa)	$\sigma_{c}$	183	122	40	13.33
Young’s Modulus (Gpa)	Ε	63.2	25.8	12.5	2.4
Poisson’s ratio	v	0.26	0.23	0.3	0.26
Cohesion (Mpa)	c	30	21	8.5	1.22
Friction angle (°)	φ	59	53	41	21
Tensile strength (Mpa)	$\sigma_{t}$	9.3	8.8	2.8	0.39
Density (Kg/m^3^)		2630	2600	2600	1450
Grain mean size (mm)		4	4	2	2

**Table 3:** Calibrated micro-properties used in the Voronoi model.

		Hard Rock ⟶ Weak Rock			
Lithology		Loc du Bonnet Granite	Augig Granite	Transjuane Sandstone	Coal
Sample rock:					
UCS (Mpa)	$\sigma_{c}$	184.5	121.4	40.5	13.1
Young’s modulus (Gpa) Poisson’s ratio	Ε v	58.9 0.254	26.197 0.232	12.93 0.3	2.27 0.263
Tensile strength (Mpa)	$\sigma_{t}$	9.8	8.6	2.6	0.38
Contact:					
Normal stiffness (Gpa)	$k_{n}$	67,500	22,432	15,000	4111.1
Shear stiffness (Gpa) Stiffness ratio	$k_{s}$	40,500 0.6	13,459 0.6	8000 0.53	3288.9 0.8
Cohesion (Mpa)	$c_{j}$	47	44	15	6.5
Friction angle (°)	$\varphi_{j}$	57.5	35	41	21
Tensile strength (Mpa)	$t_{j}$	18	11.56	3.6	0.19
Residual Cohesion Residual Friction Angle Residual Tensile Strength	$c_{jr}$ $\varphi_{jr}$ $\sigma_{tjr}$	0 30 0	0 10 0	0 5 0	0 4 0

**Table 4:** Comparison between experiment and simulation error value.

Sample	E (Gpa)	V	UCS (Mpa)	BTS (Mpa)
	Error (%)	Error (%)	Error (%)	Error (%)
LdB Granite	6.8	2.3	0.81	4.5
Augig Granite	1.54	0.86	0.49	2.6
TS Sandstone	3.44	0	1.2	1.3
Coal	−5.41	1.1	−0.17	−1

## Data Availability

Data sharing is not applicable to this article.
